# Transcriptional, Electrophysiological, and Metabolic Characterizations of hESC-Derived First and Second Heart Fields Demonstrate a Potential Role of TBX5 in Cardiomyocyte Maturation

**DOI:** 10.3389/fcell.2021.787684

**Published:** 2021-12-17

**Authors:** Arash Pezhouman, Ngoc B. Nguyen, Alexander J. Sercel, Thang L. Nguyen, Ali Daraei, Shan Sabri, Douglas J. Chapski, Melton Zheng, Alexander N. Patananan, Jason Ernst, Kathrin Plath, Thomas M. Vondriska, Michael A. Teitell, Reza Ardehali

**Affiliations:** ^1^ Division of Cardiology, Department of Internal Medicine, David Geffen School of Medicine, University of California, Los Angeles, Los Angeles, CA, United States; ^2^ Eli and Edythe Broad Stem Cell Research Center, University of California, Los Angeles, Los Angeles, CA, United States; ^3^ Molecular, Cellular and Integrative Physiology Graduate Program, University of California, Los Angeles, Los Angeles, CA, United States; ^4^ Molecular Biology Interdepartmental Doctoral Program, University of California, Los Angeles, Los Angeles, CA, United States; ^5^ Department of Bioengineering, University of California, Los Angeles, Los Angeles, CA, United States; ^6^ Department of Biological Chemistry, University of California, Los Angeles, Los Angeles, CA, United States; ^7^ Department of Anesthesiology and Perioperative Medicine, University of California, Los Angeles, Los Angeles, CA, United States; ^8^ Molecular Biology Institute, University of California, Los Angeles, Los Angeles, CA, United States; ^9^ Jonsson Comprehensive Cancer Center, David Geffen School of Medicine, University of California, Los Angeles, Los Angeles, CA, United States; ^10^ Department of Physiology, University of California, Los Angeles, Los Angeles, CA, United States; ^11^ Department of Medicine, University of California, Los Angeles, Los Angeles, CA, United States; ^12^ Department of Pediatrics, University of California, Los Angeles, Los Angeles, CA, United States

**Keywords:** first and second heart fields, single cell RNA seq, action potential, hESC-derived cardiomyocyte, maturity, regenerative medicine, metabolism

## Abstract

**Background:** Human embryonic stem cell-derived cardiomyocytes (hESC-CMs) can be used as a source for cell delivery to remuscularize the heart after myocardial infarction. Despite their therapeutic potential, the emergence of ventricular arrhythmias has limited their application. We previously developed a double reporter hESC line to isolate first heart field (FHF: *TBX5* ^+^ *NKX2-5* ^+^) and second heart field (SHF: *TBX5* ^*-*^ *NKX2-5* ^*+*^) CMs. Herein, we explore the role of TBX5 and its effects on underlying gene regulatory networks driving phenotypical and functional differences between these two populations.

**Methods:** We used a combination of tools and techniques for rapid and unsupervised profiling of FHF and SHF populations at the transcriptional, translational, and functional level including single cell RNA (scRNA) and bulk RNA sequencing, atomic force and quantitative phase microscopy, respirometry, and electrophysiology.

**Results:** Gene ontology analysis revealed three biological processes attributed to *TBX5* expression: sarcomeric structure, oxidative phosphorylation, and calcium ion handling. Interestingly, migratory pathways were enriched in SHF population. SHF-like CMs display less sarcomeric organization compared to FHF-like CMs, despite prolonged *in vitro* culture. Atomic force and quantitative phase microscopy showed increased cellular stiffness and decreased mass distribution over time in FHF compared to SHF populations, respectively. Electrophysiological studies showed longer plateau in action potentials recorded from FHF-like CMs, consistent with their increased expression of calcium handling genes. Interestingly, both populations showed nearly identical respiratory profiles with the only significant functional difference being higher ATP generation-linked oxygen consumption rate in FHF-like CMs. Our findings suggest that FHF-like CMs display more mature features given their enhanced sarcomeric alignment, calcium handling, and decreased migratory characteristics. Finally, pseudotime analyses revealed a closer association of the FHF population to human fetal CMs along the developmental trajectory.

**Conclusion:** Our studies reveal that distinguishing FHF and SHF populations based on TBX5 expression leads to a significant impact on their downstream functional properties. FHF CMs display more mature characteristics such as enhanced sarcomeric organization and improved calcium handling, with closer positioning along the differentiation trajectory to human fetal hearts. These data suggest that the FHF CMs may be a more suitable candidate for cardiac regeneration.

## Introduction

Cardiovascular disease is a leading cause of death worldwide (Benjamin et al., 2019). Due to the limited regenerative capacity of the heart, ischemic events such as myocardial infarction (MI) leads to permanent loss of CMs and replacement by scar tissue which can eventually result in heart failure (Laflamme and Murry, 2011). Despite the current standard strategies of revascularization to salvage myocardium, paradoxical effect of ischemia-reperfusion precipitates further injury of viable heart muscle (Selmer et al., 2005). Over the past 20 years, scientists have attempted many approaches to regenerate damaged heart tissue such as promoting the proliferation of endogenous CMs or direct reprogramming of resident cardiac fibroblasts into CMs (Cao et al., 2016). However, the application of these strategies has been limited due to the low proliferative capacity of adult CMs even upon exogenous stimulation as well as low efficiency of direct reprogramming (Ghiroldi et al., 2017). In recent years, pluripotent stem cell-based regenerative therapy has offered a great promise for cardiac repair. Several studies have shown that human embryonic stem cell-derived CMs (hESC-CMs) can improve cardiac function and remuscularize the heart after MI (Laflamme et al., 2007; Skelton et al., 2016a; Skelton et al., 2016b; Yanamandala et al., 2017; Liu et al., 2018). Despite the improvement in cardiac function, emergence of fatal ventricular arrhythmias has limited the application of hESC-CMs (Shiba et al., 2016). Electrophysiological studies on transplanted hESC-CMs also provide evidence that graft-induced arrhythmias may result from pacemaker-like activity rather than abnormal conduction (Liu et al., 2018).

Our group recently introduced a new approach to generate heart field-specific CMs from differentiating hESCs which can be used to provide insight into the emergence of fatal arrhythmias post-transplantation (Pezhouman et al., 2021). Using a double reporter system comprising of early cardiac transcription factors *TBX5* and *NKX2-5*, we were able to isolate first heart field (FHF) (*TBX5* ^*+*^ */NKX2-5* ^*+*^) and second heart field (SHF) (*TBX5* ^*-*^ */NKX2-5* ^*+*^) CMs while excluding pacemaker-like cells (*TBX5* ^*+*^ */NKX2-5* ^*-*^). Investigators have shown that within the heart, *TBX5* is predominately expressed in the primitive posterior heart tube, marking progenitors of the LV and atria, corresponding to the FHF (Bruneau et al., 1999; Liberatore et al., 2000; Bruneau et al., 2001; Sizarov et al., 2011; Xie et al., 2012; Später et al., 2013; Steimle et al., 2018). Our study showed that delineation of hESC-derived heart-field specific CMs using TBX5 roughly mirrored the previous *in vivo* models. Prior studies (Lundy et al., 2013; Lewandowski et al., 2018) have shown that prolonged *in vitro* culture of stem-cell derived CMs may lead to the maturation of their structural and contractile properties to a more adult-like phenotype. Interestingly, we found significant differences in electrophysiological properties of FHF- and SHF-like CMs. Given the persistent delay in maturation of SHF-like CMs, we hypothesized that these intrinsic differences between FHF- and SHF-like populations may be driven by early expression of TBX5 and its downstream effects on biological processes involved in cardiac maturation. The role of TBX5 in early cardiac development and chamber-specificity is well-studied; however, its effects on later stages of development and maturation of CMs remains unknown.

In this study, we used a stepwise approach to identify and validate underlying biological processes that lead to the phenotypical differences observed between FHF- and SHF-like CMs. Unsupervised differential gene expression analyses revealed four main biological processes including muscle cell development, oxidation phosphorylation, response to wounding, and calcium handling. We next conducted relevant biological and/or functional assays to validate these biological processes, including Seahorse Assay, atomic force microscopy, and optical mapping. We also performed pseudotime analyses of our FHF- and SHF-like CMs compared to human fetal CMs to determine where hESC-derived CMs are positioned along the developmental trajectory. Our study reveals that not only *TBX5* expression is important for early cardiac development and chamber specification, but it may also play a role in later stages of differentiation and cardiac maturation by fine-tuning sarcomeric organization and calcium handling.

## Methods

### Differentiation of Double Reporter hESC Line

We have previously generated hESC *TBX5-TdTomato* ^*+/W*^/*NKX2-5* ^eGFP/W^ double reporter using a HES3-*NKX2-5* ^*eGFP/W*^ line generously provided by E. Stanley and A. Elefanty (Monash University, Victoria, AU) (Elliott et al., 2011; Pezhouman et al., 2021). Monolayer cardiac differentiation was achieved using small molecule inhibitors of GSK3 and Wnt. HES3-*TBX5-TdTomato* ^*+/W*^/*NKX2-5* ^eGFP/W^ cells were expanded on Geltrex (Gibco™, A1413202) to 70–85% confluency then harvested as a single-cell suspension using Accutase [Gibco™, A1110501)] and resuspended in mTeSR plus (Stem Cell™ Technologies, 05826) containing 10 µM ROCK inhibitor Y-27632 (Tocris Biosciences, 1,254). Cells were counted using a Countess II Automated Cell Counter (Countess™, AMQAX1000) and re-plated onto Geltrex coated plates at 1 × 10^5^ cells/cm^2^ in mTeSR™ Plus containing 10 µM ROCK inhibitor Y-27632 (day -2 of differentiation). At day -1 media was changed to mTeSR™ Plus. At day 0 media was changed to RPMI (Gibco™, 11875093) containing B-27™ supplement, minus Insulin (Gibco™, A1895601) containing CHIR99021 (Tocris, 4,423) (10 µM). After 24 h (day 1) media was aspirated and replaced with RPMI B27 minus Insulin until day 3. On day 3 of differentiation media aspirated and replaced with RPMI B27 minus Insulin containing 5 μM IWP2 (Tocris, 3,533). At day 5, media was changed to RPMI B27 minus Insulin until day 7 when media was switched to RPMI containing B-27™ supplement (Gibco™, A3582801). Cells were maintained in this media and changed every 3 days thereafter.

### Flow Cytometry and Cell Sorting

Differentiated hESCs were dissociated with Accutase™ (STEMCELL Technology, 07920) for 3–5 min at 37°C to form a single cell suspension. Cells were resuspended in FACS buffer (2% FBS, 1% BSA, 2 mM EDTA) containing 10 µM ROCK inhibitor Y-27632 and DAPI. Cells were sorted using a FACS-ARIA-H (BD Biosciences) into RPMI containing B-27™ supplement (Gibco™, A3582801) with 10 µM ROCK inhibitor Y-27632. FACS data were analyzed using FlowJo software (Tree Star Inc.).

### Bulk RNA-Sequencing

FHF (TdTomato^+^/GFP^+^), SHF (TdTomato^−^/GFP^+^) cells were FACS sorted on day 20 of differentiation. RNA of the FHF and SHF cells was isolated using TRIZOL LS Reagent (Invitrogen™, 10296028), RNeasy Micro Kit (Qiagen, 74004) was used for purification. The quality of the RNA was assessed by Agilent 2,200 Tapestation. For library preparation, total RNA was fragmented and subjected to cDNA conversion, adapter ligation, and amplification using KAPA Stranded RNA-Seq Library Preparation Kit (KAPA Biosystems, KK8502) according to the manufacturer’s instructions. The final library was quantified using Agilent 2,100 Bioanalyzer to evaluate its integrity. The deep sequencing for 2 × 150 bp paired-end reads was performed using Illumina Novaseq 6,000. For sample analysis, RNA-seq data was mapped to the reference genome (GRCh38) with OLego version 1.1.5 and normalized by using TPM (Transcripts per millions) analysis. Total number of reads mapped to a known transcript annotation was estimated using featureCounts version v1.5.0-p2. Expression levels for each transcript were determined by normalizing the counts returned by featureCounts using custom Perl scripts. Normalized expression levels for each transcript were determined by transforming the raw expression counts to TPM following log2 scaling. Gene Ontology (GO) enrichments were computed using Metascape (Zhou et al., 2019). RStudio was used to run custom R scripts to generate boxplots and heatmaps using “heatmaply” package.

### Single Cell RNA Sequencing of Human Fetal Hearts and hESC-Derived CMs

Human fetal heart 6, 10, and 17 weeks of gestation was digested into single cell suspension using Collagenase II (Worthington, LS004176, 0.45 mg/ml) and Pancreatin (Sigma, P3292-25G, 1 mg/ml). Day 20 FHF and SHF cells were digested into single cell suspension using TrypLE. Single cells were captured using the 10X Genomics Chromium Single Cell v.2 platform. cDNA libraries derived from the FHF and SHF were independently generated and sequenced on the Illumina NextSeq, generating 490 million reads of FHF-derived samples and 466 million reads of SHF-derived libraries of which >97% passed quality control**.** cDNA libraries from human fetal heart were sequenced with the Illumina NovaSeq. Digital expression matrix was generated by de-multiplexing, barcode processing, and gene unique molecular index counting using the Cell Ranger v3.0 pipeline and the GRCh38 reference genome. Cells that express less than 200 genes, and genes detected in less than 3 cells were filtered out. The Seurat 4.0.4 R toolkit (Hao et al., 2021) for single cell genomics was used to analyze sequencing results. Downstream analysis was restricted to cells associated with at least 3,000 unique molecular identifiers (UMIs). For identification of cell clusters in the human fetal heart (*TNNT2*, *MYH7*) *(COL1A1, DDR2)*, and (*PECAM1, CDH5*) were used to identify cardiac, fibroblast, and endothelial cell clusters, respectively. The FeaturePlot, BoxPlot, and ViolinPlot functions of Seurat were used to visualize genes of interest.

### Monocle Pseudotime Analysis

The Seurat object file was converted into a CellDataSet (CDS) for further analysis using the Monocle 3 software (Trapnell et al., 2014). Cell clusters and trajectories were visualized using the standard Monocle workflow. The first 20 PCs were used for pre-processing (preprocessCDS). Then, these lower-dimensional coordinates were used to initialize a nonlinear manifold learning algorithm implemented in Monocle 3 called “UMAP” (*via* reduceDimension; Becht et al., 2018). This allows us to visualize the data in two dimensions. The “cluster_cells” function was used to identify clusters and then “learn_graph” was used to learn the sequence of gene expression changes of each cell to generate an overall trajectory. To mark the root of the pseudotime, we used the “order_cells” function to assign the starting root of pseudotime. Gene expression along pseudotime were plotted using the “plot_genes_in_pseudotime” function, with “color_cells_by” parameter set at “ID.”

### TBX5 and NKX2-5 Binding Simulation

To test whether TBX5 and NKX2-5 preferentially bind to FHF and SHF gene promoters, we first empirically defined gene markers for each cell population using the FindMarkers() function in Seurat with the following parameters: ident.1 = “TBX5+ CMs,” ident.2 = “TBX5- CMs,” and min.pct = 0.3. For this simulation, we defined FHF genes as the marker subset with avg_log2FC > 0, and SHF genes as the subset with avg_log2FC < 0. As a negative control, we randomly selected 10 subsets of 150 genes (excluding FHF and SHF genes) from the count matrix. We then downloaded fetal (E12.5) *TBX5* and *NKX2-5* bioChIP-seq peak sets from online dataset (Akerberg et al., 2019) and determined mm9 promoter coordinates of murine orthologs of the FHF, SHF, and random gene subsets. To test whether regions of *TBX5* or *NKX2-5* binding significantly overlap with our gene subsets, we used hypergeometric tests as in (Chapski et al., 2021). Because we performed a total of 12 statistical tests during each transcription factor simulation (FHF, SHF, and 10 random gene sets), we corrected the *p*-values using the Benjamini-Hochberg (Benjamini and Hochberg, 1995) method in R.

### Immunocytochemistry

Immunocytochemical staining were performed on cells seeded onto Geltrex coated optical tissue culture chamber (Thermo Scientific Lab-Tek II Chamber Slide System, 154,526). Cells were fixed in 4% paraformaldehyde (PFA) in PBS for 15 min at RT followed by PBS washings. For staining, fixed cells were permeabilized with 0.2% Triton X-100 (Fisher Bioreagents, BP151) in PBS for 10 min prior to blocking non-specific binding with 10% normal serum in PBST (PBS with 0.1% Tween-20 (MP Biomedicals, 11TWEEN201) for 30 min. Cells were incubated with primary antibodies at 4°C overnight and then stained with secondary antibodies at room temperature for 1 h and mounted with VECTASHIELD Antifade Mounting Medium with DAPI (Vector Laboratories, H-1200). The stained cells were imaged with a Leica TCS SP5 microscope using LAS X software (Leica Biosystems) or an LSM 880 with Airyscan Confocal Microscope using ZEN software (Carl Zeiss Microscopy). The following primary antibodies were used: Rabbit anti-Cardiac Troponin T (Abcam, ab45932,1:400), Mouse anti-cardiac ACTN2 (Sigma, A7811, 1:300), Rabbit anti-TOMM20 (Abcam, ab2043078, 1:1,000), Mouse anti-dsDNA (Abcam, ab470907, 1:1,000).

### OCR and ECAR Measurements

Oxygen consumption rate and extracellular acidification rate were quantified using an Agilent Seahorse XFe96 Extracellular Flux Analyzer. 2 × 10^4^ cells were seeded on each well of a V3 96-well plate (Agilent, Cat#101085-004) and cultured for 2–3 days prior to analysis. The Agilent Seahorse mitochondrial stress test was used to measure basal OCR and ECAR as well as OCR and ECAR following sequential addition of the electron transport chain inhibitor drugs oligomycin, carbonyl cyanide-p-trifluoromethoxyphenylhydrazone (FCCP), and antimycin A. Results were normalized to cell count and analysed using the Agilent Wave 2.6.2 software package.

### Quantitative Phase Microscopy and Cellular Motion Measurements

Quantitative phase images on FHF and SHF cells were obtained with 20 × 0.4 numerical aperture objective lens on an Axio Observer Z1 inverted microscope with a temperature and CO2 regulated stage-top cell incubation chamber (Zeiss). Quantitative phase data was obtained with a SID4Bio (Phasics) quadriwave lateral shearing interferometry (QWLSI) camera (Bon et al., 2009; Zhang et al., 2013) while fluorescence images were obtained on an EM-CCD C9100 camera (Hamamatsu Photonics). A 660 nm centred wavelength collimated LED (Thorlabs) was used as the *trans*-illumination source for QPM and an X-Cite Series 120 Q (Lumen Dynamics) source for fluorescence imaging. Image collection occurred every 10 min for over 2 d 14—20 imaging locations containing cells plated with sufficient spacing to enable automated image processing and biomass segmentation. All image processing was performed using custom MATLAB (MathWorks) scripts. Cells and clusters were identified and segmented using a local adaptive threshold based on Otsu’s method (Otsu, 1979) with particle tracking code based on Grier *et al* (Zangle et al., 2013; Nguyen et al., 2020)*.* Net positional displacement of cells were calculated based on the differences in cell position in tracks in one frame compared to the next while percent mass fluctuations was calculated based on the difference in mass distribution of cells normalized with regards to cellular mass from one frame compared to the next.

### Atomic Force Microscopy

We have employed a combination of atomic force microscopy (AFM)/confocal microscopy techniques to determine the mechanical properties of FHF and SHF cells. The nanoindentation and data acquisition were performed using a JPK Nanowizard 4A Atomic force microscopy combined with a Zeiss LSM 510 confocal microscope. The stage is arranged so that we can independently control the AFM cantilever, sample, and confocal microscope objective. Using the confocal microscope, we verified that the cells chosen for the nanoindentation experiment are the FHF (*TBX5* ^*TdT+*^ */NKX2.5* ^*eGFP+*)^ or the SHF (*TBX5* ^*TdT-*^ */NKX2-5* ^*eGFP+*^).

To do the nanoindentation experiment, a cell culture dish was mounted on the AFM stage (temperature = 36.5°C during the experiments) and the cantilever tip approaches the sample from a few microns above the sample (maximum applied force = 3 nN, indentation speed = 2 μm/s). A soft AFM probe (spring constant k = 0.286 nN/nm, Bruker, NY, United States) with a spherical tip of 10 µm diameter was used for this experiment. Indentation and retraction of cantilever was plotted and processed using JPK analysis software. The built-in Hertz/Sneddon model fitting tool of the JPK software was used to compute Young’s modulus values. Hertz model for a spherical tip was calculated using following equation.

$$\text{F}=\;\frac{4\sqrt{\text{R}}}{3}\frac{E}{1-\upsilon^{2}}\delta^{3/2}$$

(F = indentation force, R = radius of the cantilever tip, E = Young’s modulus, υ = poison ration (0.5 in our experiment) and δ = indentation depth). The average stiffness of five local regions on top of the nucleus in each cell, is reported as the stiffness of the cell.

### Monolayer Optical Mapping

FACS-isolated cells were suspended in RPMI B27 supplemented with ROCK inhibitor Y-27632 (10 µM) at 20-22 × 10 (Yanamandala et al., 2017) cells/ml. Drops of 25 µl of this cell suspension were applied to Geltrex-coated 5 mm coverslips (5 × 10 (Ghiroldi et al., 2017) cells/coverslip). The cells were incubated in the 25 µl volume for 8–12 h to facilitate cell attachment. Once spontaneous contractions were observed, cells were stained with voltage-sensitive dye, Di-8-ANEPPS (Invitrogen, D3167, 40 µM) and washed with normal Tyrode solution three times (Pezhouman et al., 2014; Pezhouman et al., 2015; Pezhouman et al., 2018; Pezhouman et al., 2021). Optical AP recording were made using MiCAM-Ultima CMOS camera at 500 frames per second (fps). Spontaneously occurring APs were recorded and APD_30_ and APD_80_ were measured using BV-Ana (1,604) software.

### Data and Software Availability

The RNA-sequencing data has been deposited in the SRA repository with BioProject accession numbers PRJNA773814.

### Study Approval

The collection and use of human fetal material were carried out following federal and local approval, including the United States Institutional Review Board (IRB 11-002504) to the Translational Pathology Core Laboratory of the Department of Pathology and Laboratory Medicine at UCLA. Cardiac tissues from human embryos were collected with informed consent following surgical termination of pregnancy and staged immediately by stereomicroscopy according to the Carnegie classification. All identifiers were removed before obtaining the samples.

### Statistics

All data are represented as individual values. Due to the nature of the experiments, randomization was not performed, and the investigators were not blinded. Statistical significance was determined by using student’s t test (unpaired, two-tailed) in GraphPad Prism 8 software. Results were significant at *p* < 0.05 (*), *p* < 0.01 (**), *p* < 0.001 (***), and *p* < 0.0001 (****). All statistical parameters are reported in the respective figures and figure legends. All error bars are depicted as SEM.

## Results

### Biological Process Analyses Reveal Enrichment of Structural, Metabolic, and Calcium Handling Pathways Within FHF-Like Compared to SHF-Like CMs

**FIGURE 1:**
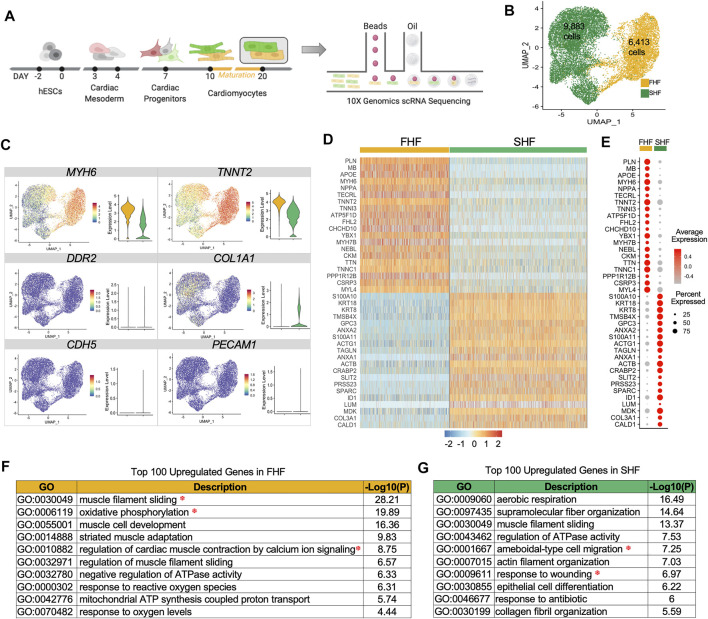
Single cell RNAseq of first and second heart field hESC-derived cardiomyocytes. **(A)**, Schematic representation of lineage commitment of hESCs into cardiomyocytes for single cell capture using 10X Genomics. **(B)**, Unsupervised clustering of FHF (yellow) and SHF (green) populations projected on a two-dimensional UMAP. **(C)**, UMAP and boxplots showing gene expression quantification for the cardiac genes (*MYH6*, *TNNT2*), fibroblast genes (*DDR2*, *COL1A1*), and endothelial genes (*CDH5, PECAM1*). **(D)**, The expression heatmap of top 20 differentially expressed genes in FHF and SHF. **(E)**, DotPlot analysis (average and percent expression) of top 20 differentially expressed genes in FHF and SHF populations. **(F)**, Gene Ontology analysis of the top 100 upregulated genes enriched in Day 20 FHF-like CMs. **(G)**, Gene Ontology analysis of the top 100 upregulated genes enriched in Day 20 SHF CMs.

We used our previously generated HES3-*TBX5-TdTomato* ^*+/W*^ */NKX2-5* ^*eGFP/W*^ double reporter line (Pezhouman et al., 2021) to isolate FHF (*TBX5* ^*+*^ */NKX2-5* ^*+*^) and SHF (*TBX5* ^*-*^ */NKX2-5* ^*+*^) CMs using a monolayer cardiac differentiation protocol (GSK3 inhibitor/Wnt inhibitor (GIWI)) (Supplementary Figures S1A,B). Fluorescent imaging showed clusters of cells that express both TdTomato and GFP (FHF) or solely GFP (SHF) (Supplementary Figure S1C). Fluorescence activated cell sorting (FACS) was used to isolate these two distinct populations using their respective fluorescent markers (Supplementary Figure S1D). We had previously shown that FHF-like CMs exhibit longer action potential duration compared to SHF, suggesting a potential role of TBX5 expression in orchestrating downstream signaling pathways that may lead to these functional and phenotypic differences. Here, we use the 10X Genomics (10X Genomics, 2020) and Seurat toolkits (Hao et al., 2021) to transcriptionally profile FACS-isolated FHF and SHF cells to unravel the contribution of TBX5 expression to cardiomyocyte maturation. After removal of low-quality cells, we obtained 9,883 SHF and 6,413 FHF single-cell transcriptomes for downstream analyses (Figures 1A,B). Principal component analysis revealed that PC1 separated these 2 cell populations, with correlated genes belonging to the structural proteins, including *MYH6*, *TNNT2*, and *TTN* (Supplementary Figures S1E,F). Because FHF-like CMs were selected based on expression of *TBX5*, we asked whether promoters of genes preferentially expressed in FHF-like CMs (FHF genes) have higher *TBX5* occupancy compared to that of SHF gene promoters. To test enrichment, we downloaded a murine *TBX5* bioChIP-seq peak set from fetal (E12.5) ventricles (Akerberg et al., 2019) and determined binding enrichment at murine orthologs of FHF and SHF genes using hypergeometric tests. Interestingly, both FHF and SHF genes are significantly bound by *TBX5* (corrected *p*-values of 9.71 × 10^–12^ and 2.18 × 10^–7^ for FHF and SHF genes, respectively) (Supplementary Figure S1G). Because both FHF- and SHF-like CMs express *NKX2-5* at some point in development, we also examined a murine *NKX2-5* bioChIP-seq dataset from the same study (Akerberg et al., 2019) and found significant enrichment of NKX2-5 occupancy at both promoter sets (corrected *p*-values of 2.21 × 10^–22^ and 9.50 × 10^–12^ for FHF and SHF gene promoters, respectively) (Supplementary Figure S1H). Notably, we did not observe enrichment of *TBX5* and *NKX2-5* at the promoters of random gene sets (Supplementary Figures S1G,H). Taken together, these data suggest that the FHF and SHF populations represent myocyte-like cells that express a set of genes preferentially bound by *TBX5* and *NKX2-5*.

To test the robustness of our approach in capturing pure CMs in both populations, we examined the expression of known cardiac cell type markers such as *MYH6* and *TNNT2* (cardiomyocyte), *DDR2* and *COL1A1* (fibroblast), and *CD31* and *CDH5* (endothelial). Only CM markers were expressed in these two populations, with higher expression of *MYH6* and *TNNT2* in FHF cells (Figure 1C). Heatmap analyses of the top 20 differentially expressed genes in FHF- and SHF-like CMs showed uniformity of gene expression within each population as well as distinct gene profiles between the two populations (Figure 1D). DotPlot analysis revealed that certain genes such as *TNNT2*, *MYL4*, *TTN*, and *TNNC1* have a high percent expression within both populations (i.e., percent of the cells that express the gene of interest); however, average expression levels are higher in FHF (i.e., average expression of gene of interest across all cells). On the other hand, genes such as *PLN*, *MB*, and *TNNI3*, exhibit both higher percent and average expression in FHF-like compared to SHF-like CMs (Figure 1E).

To unravel the biological processes enriched in FHF- and SHF-like CMs, we performed gene ontology (GO) analysis of the top 100 upregulated genes within each population using Metascape (Zhou et al., 2019). The top GO term categories within the FHF population revealed three main categories: muscle structure (muscle filament sliding, muscle cell development, striated muscle adaptation), metabolism (oxidative phosphorylation, mitochondrial ATP synthesis coupled proton transport), and calcium handling (regulation of cardiac muscle contraction by calcium ion signaling) (Figure 1F). Interestingly, while SHF-like CMs shared structural (supramolecular fiber organization) and metabolism (aerobic respiration) related pathways, they were also distinctively enriched in cell migration and wound response pathways (Figure 1G). Given that structural, metabolic, and calcium handling categories enriched in the FHF population are important aspects of cardiac physiology, we further focused on the genes assigned to these pathways to better characterize the differences between FHF and SHF populations. This investigation would address our overall goal of determining whether *TBX5* expression in cardiac cells plays a role in mediating downstream signaling pathways leading to cardiac maturation.

### Enhanced Sarcomeric Organization and Cellular Stiffness Within FHF-Like CMs

**FIGURE 2:**
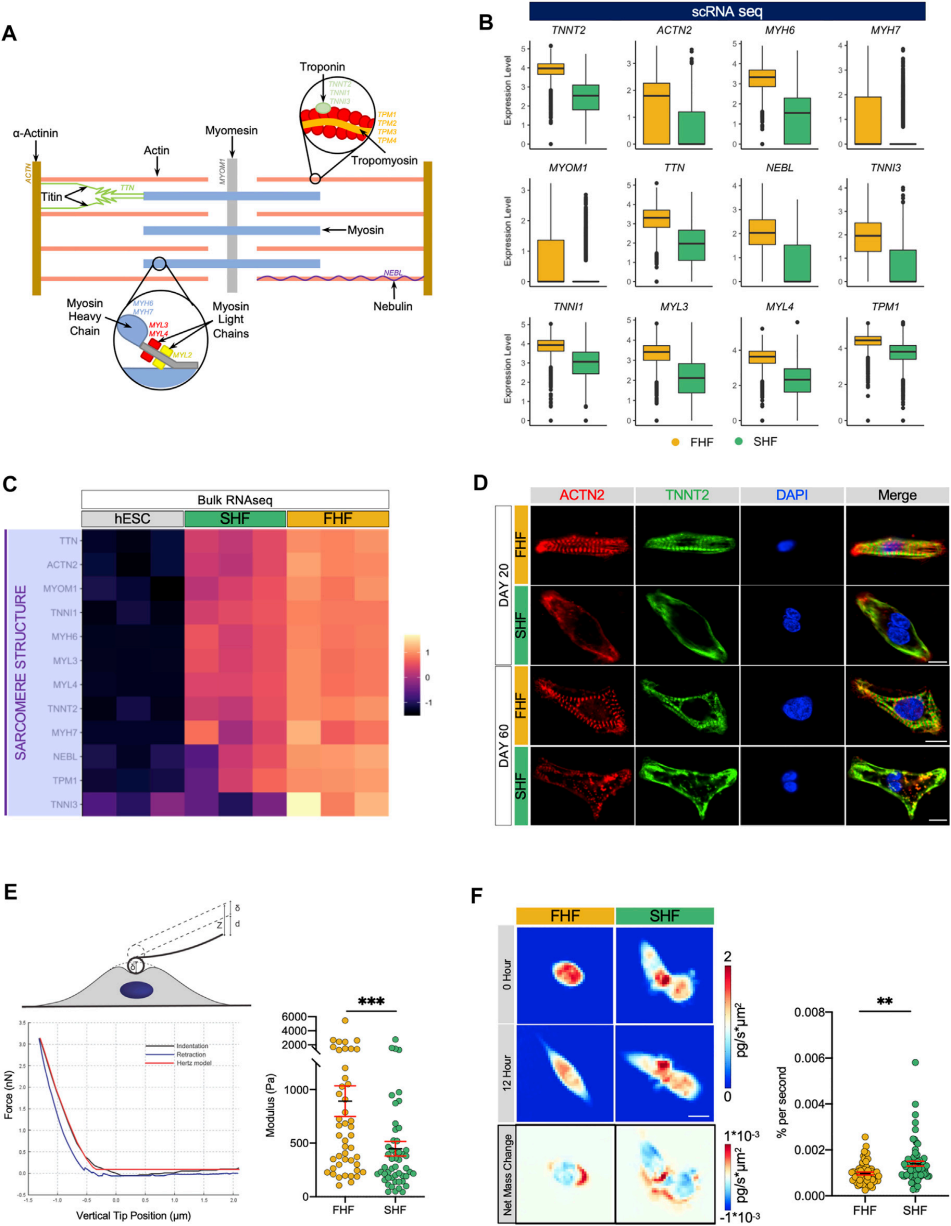
Differences in sarcomeric organization, cellular stiffness, and migration between FHF- and SHF-like CMs. (**A)**, Schematic of important structural and regulatory components of a cardiac sarcomeric unit. **(B)**, Boxplots of structural genes extracted from GO pathway “GO:0030049: muscle filament sliding” with higher average expression in FHF compared to SHF CMs. **(C)**, Heatmap of cardiac sarcomeric structural genes extracted from GO:0030049 using bulk RNA sequencing data of hESCs and FHF- and SHF-like CMs at Day 20. **(D)**, Immunocytochemistry of Day 20 **(top)** and Day 60 **(bottom)** FHF- and SHF-like CMs for cardiac structural markers ACTN2 and TNNT2. Nuclei were visualized with DAPI, scale = 10 μm. **(E)**, **(top)** Schematic of experimental indentation set-up. A cell is indented by an AFM cantilever with a spherical probe. The vertical position of the cantilever is moved by z; the cantilever bends by d; the cell is indented by δ; z = d + δ **(bottom left)** Example of a force vs. indentation (black) and retraction (blue) curves obtained by atomic force microscopy. The indentation data was fit using the Hertz model (red). **(right)** Dot plot of FHF (*n* = 58) and SHF (*n* = 68) Young’s Modulus calculated from atomic force microscopy. **(F)**, **(top left)** Quantitative phase mass distribution images of FHF- and SHF-like CMs at the initial time of imaging and 12 h after **(bottom left)** Images showing the spatial redistribution of mass over the course of the 12-h period for the FHF- and SHF-like CM clusters shown above. Dot plot of FHF (*n* = 58) and SHF (*n* = 68) clusters’ magnitude of net mass change derived from the averages of QPI mass redistribution images.

A prominent and unique feature of cardiac muscle is its sarcomeric strucutre. Sarcomeres give cardiac muscle their striated appearance and are the repeating segments that make up muscle filaments. In addition to actin and myosin, other proteins such as α-actinin (*ACTN*), myomesin (*MYOM1*), nebulin (*NEBL*), and titin (*TTN*) are important contributors in forming organized sarcomeric structures that are crucial for CM contractile function (Figure 2A). To determine structural differences between FHF and SHF populations, we compared the expression level of certain structural genes extracted from the GO pathway “GO:0030049: muscle filament sliding” from our single cell RNA sequencing dataset. Boxplot analysis revealed that expression of key structural-related genes such as *TTN, NEBL, MYOM1, MYH6, MYH7*, and *TNNT2* are higher within the FHF compared to SHF population (Figure 2B). Current scRNA-seq technology may have technical biases that if not correctly adjusted, can lead to severe type I error in differential expression analysis (Jia et al., 2017). To avoid this, we tested the average expression level of sarcomeric structure-related genes from GO:0030049 using bulk RNA sequencing (bulk RNA-seq), which were in alignment with our scRNA-seq data (Figure 2C), although some genes were more highly expressed in SHF **(** Supplementary Figures S3A,B). As gene-protein relationships are not always directly correlated, we next examined the structural organization of FHF and SHF populations using immunocytochemistry. Day 20 FACS sorted cells were replated and doubled stained with ACTN2 and TNNT2 (Figure 2D, top half). Interestingly, immunocytochemistry (ICC) of FHF-like CMs revealed enhanced alignment and organization of sarcomeres when compared to SHF populations. Studies have suggested that prolonged culture of hESC-derived CMs may promote their sarcomeric organization. To ensure that the structural differences observed between FHF and SHF are due to their intrinsic properties and not a time-dependent delay in the maturation of SHF CMs, we cultured both population of CMs for an additional 40 days. Although SHF-like CMs showed improvement in sarcomeric organization on Day 60 compared to Day 20, the extra time in culture did not result in similar extent of structural organization as FHF-like CMs (Figure 2D, bottom half). Previous studies have shown a direct correlation between sarcomeric structure organization and cellular stiffness (Akiyama et al., 2006). To investigate the surface rigidity of our heart field-specific CM populations, we used atomic force microscopy (AFM), a technique that employs a nanoscale tip to measure the tip-sample interaction force as a surrogate readout of cellular stiffness. Measurement of FHF-(*n* = 46) and SHF-like CMs (*n* = 51) showed a significantly higher Young’s Modulus in FHF-like compared to SHF-like CMs, illustrating a more cellular stiffness property of FHF-like CMs (Figure 2E).

Interestingly our GO term analysis of upregulated genes within SHF population revealed enrichment of migratory related pathways such as (GO:0001667: ameboidal-type cell migration and GO:0009611: response to wounding). Boxplot analysis revealed that expression of key genes associated with migratory processes such as *SLIT2, COL3A1, SPARC, ANAXA1, TUBA1A* are higher within the SHF compared to FHF population (Supplementary Figures S3C,D). To investigate intrinsic migratory characteristics of these two populations, we used quantitative phase microscopy (QPM). Time-lapsed images of quantitative phase data of FHF (*n* = 58) and SHF (*n* = 68) allow for measurements of cellular mass and motion. The identity of these cells was tracked using integrated fluorescent microscopy (Supplementary Figure S3E). Although there was no significant difference in mass nor in mass accumulation between these 2 cell types, images of mass redistribution from QPM revealed greater internal mass redistribution for the SHF CMs over those of the FHF-like CMs demonstrated by the darker blue and red regions in SHF CMs. Additionally, population average values showed that the SHF had significantly greater (*p* < 0.01) internal mass motion than cells of the FHF (Figure 2F; Supplementary Video S1). This greater mass movement within SHF cells indicates greater mass motility in line with our observations from the Young’s Modulus data that SHF-like CMs are softer than FHF-like CMs *via* links between mass fluctuations and biophysical stiffness (Nguyen et al., 2020).

### Optical Mapping Reveals Longer Phase 2 (Plateau) of Action Potentials Within FHF-Like Compared to SHF-Like CMs

**FIGURE 3:**
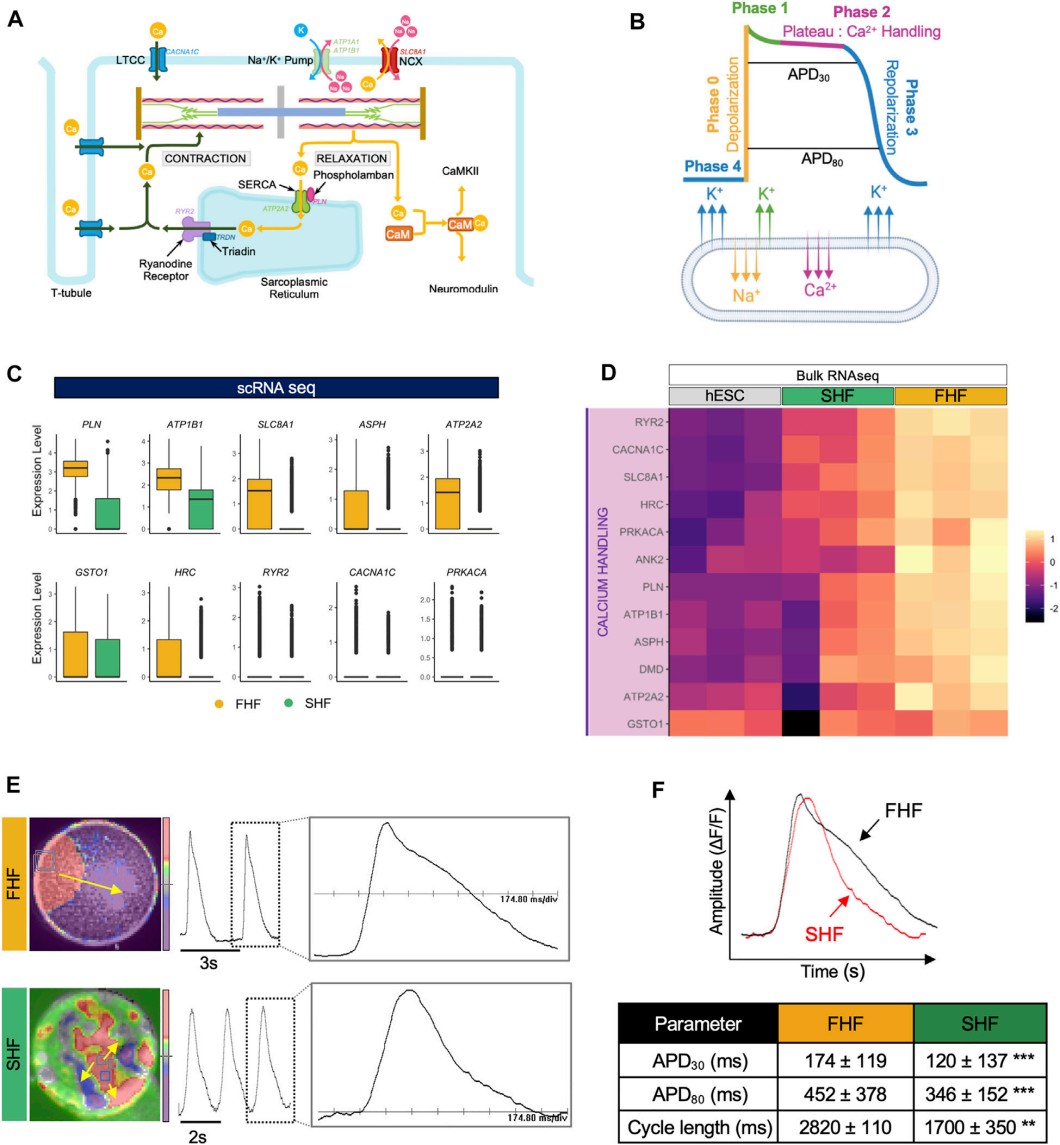
Optical mapping reveals longer phase 2 (plateau) of action potentials within FHF-like compared to SHF-like CMs. **(A)**, Schematic of calcium homeostasis within CMs. **(B)**, Schematic of action potential waveform separated by phase with corresponding ion flux. **(C)**, Boxplots of calcium handling-related genes extracted from GO term “GO:0010882: regulation of cardiac muscle contraction by calcium ion signaling” with higher average expression in FHF compared to SHF CMs. **(D)**, Heatmap of genes from panel C using bulk RNA sequencing data of hESCs and FHF- and SHF-like CMs at Day 20. **(E)**, Snapshot of optical mapping showing spontaneous APs which propagate across monolayer of FHF **(top)** and islands of SHF CMs **(bottom)**. The yellow arrow denotes direction of AP propagation. Single waveform magnified from dashed box inset scaled to 174.80 ms/div. **(F)**, **(top)** Superimposed APs from FHF- and SHF-like CMs showing longer APD in FHF-like CMs (bottom) Average values of APD_30_, APD_80,_ and cycle length measured from FHF-like (*n* = 7,776) and SHF-like (*n* = 8,464) CMs.

Our GO term analyses revealed calcium ion signaling as one of the pathways enriched in FHF-like CMs. Indeed, calcium is a critical regulator of CM function, forming a link between electrical signals and mechanical contraction of CMs, a process known as excitation-contraction coupling (EC coupling). Given their critical role in maintenance of normal cardiac rhythm, calcium ions are tightly regulated by a sophisticated machinery which consists of different channels [L-Type Ca (LTCC), ryanodine receptors (RYR2), and sarco/endoplasmic reticulum Ca2+ ATPase (SERCA)] as well as regulators (phospholamban (PLN), triadin (TRDN), and calcium calmodulin-dependent protein kinase 2 (CaMKII)) (Figure 3A). An action potential (AP) waveform represents the net influence of different ions, such as Na^+^, Ca^2+^, and K^+^ channels, of which the plateau portion of the AP reflects the Ca^2+^ handling machinery function and coordination (Figure 3B).

To determine calcium signaling differences between FHF and SHF populations, we compared the expression level of key calcium machinery genes extracted from the GO term “GO:0010882: regulation of cardiac muscle contraction by calcium ion signaling” using our single cell RNA sequencing dataset. Boxplot analysis revealed that expression of key genes such as *PLN*, *ATP2A2* (SERCA), and *RYR2* (ryanodine receptor) are higher within the FHF compared to the SHF population (Figure 3C). These findings were confirmed in our bulk RNA-seq dataset (Figure 3D). Expression of genes such as *CALM1*, *CALM2*, and *CALM3* (calmodulin family) were also higher in SHF compared to FHF. Interestingly, bulk RNA-seq analysis of those genes shows high expression in undifferentiated hESCs as well (Supplementary Figures S3A,B).

To determine whether differences in calcium handling gene expression led to functional changes in action potential (AP) waveforms, particularly in the plateau phase, we used an optical mapping technique that enables the capture of electrical activity of thousands of cells simultaneously. FACS-isolated FHF- and SHF-like CMs were re-plated to form monolayers which were then optically mapped using voltage dye. FHF CMs showed uniform AP whereas, SHF CMs formed islands of cells in which APs propagated independently, but uniformly, in each island (Pezhouman et al., 2021). Spontaneous APs were recorded from FHF-like (Figure 3E, top) and SHF-like (Figure 3E, bottom) CMs**.** APD_30_ and APD_80_ distributions of FHF (*n* = 7,776) and SHF (*n* = 8,464) were measured and summarized in (Supplementary Figures S3C,D). APD_30_ (representative of net function of Ca^2+^ handling machinery) and APD_80_ (representative of Ca^2+^ handling and repolarization) analyses revealed that FHF-like CMs have significantly longer APD_30_ (45% increase) and APD_80_ (30% increase) durations compared to SHF population. Cycle length (representative of depolarization, repolarization, and diastolic interval) analysis revealed longer cycle length in FHF compared to SHF population (Figure 3F). These results suggest the presence of a more mature calcium handling machinery in FHF-like CMs as supported by our scRNA-seq results and enhanced sarcomeric structure observation, further supporting the likelihood that FHF-like CMs are more mature than SHF CMs.

### FHF- and SHF-Like CMs Share Nearly Identical Mitochondrial Respiratory Profiles

**FIGURE 4:**
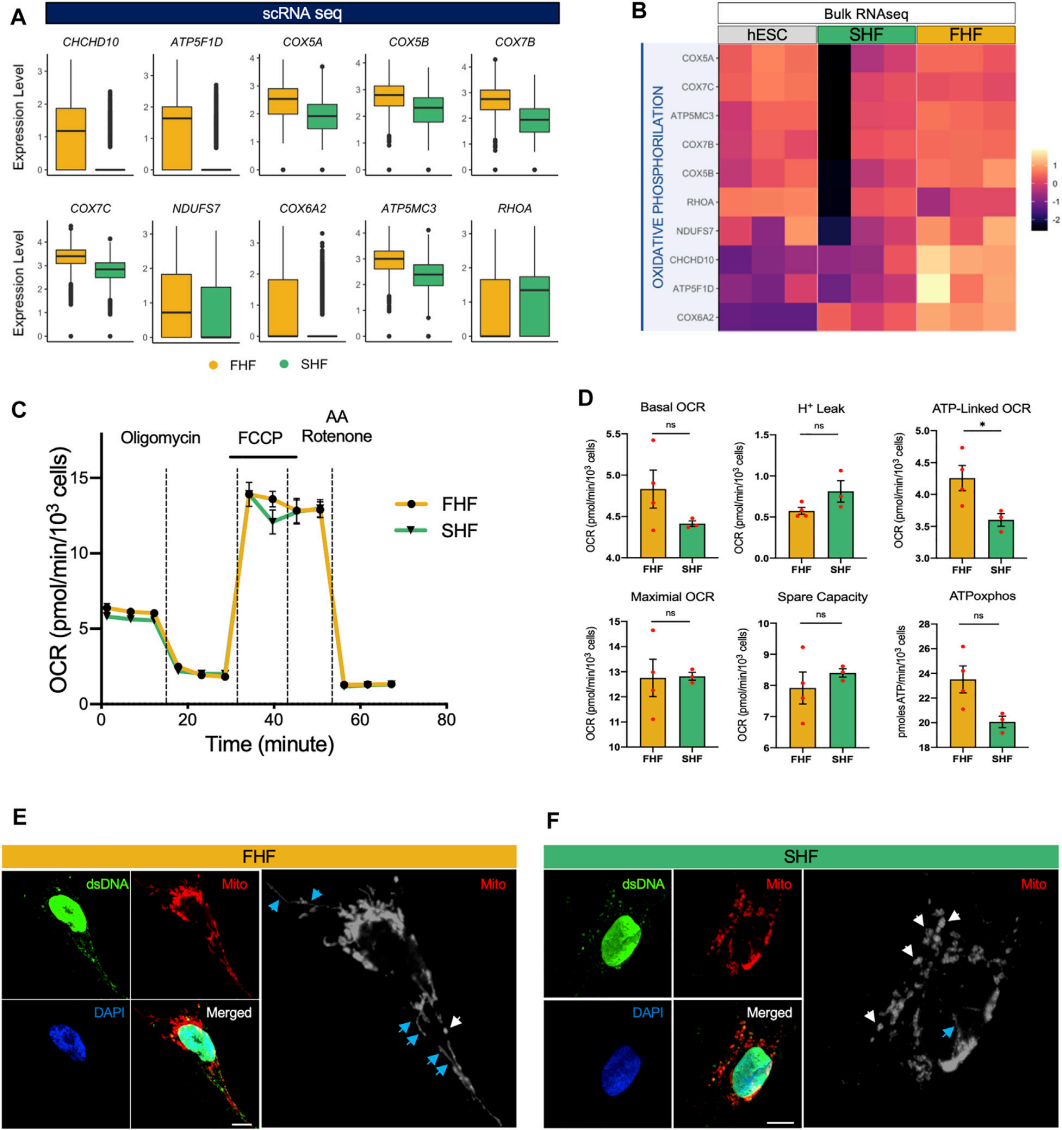
Transcriptional and functional analyses of oxidative phosphorylation and mitochondrial networks in FHF- and SHF-like CMs. **(A)**, Boxplots of oxidative phosphorylation-related genes extracted from GO term “GO: 0006119: oxidative phosphorylation” with higher average expression in FHF compared to SHF CMs. **(B)**, Heatmap of genes from panel A using bulk RNA sequencing data of hESCs and FHF- and SHF-like CMs at Day 20. **(C)**, Seahorse Extracellular Flux Analysis comparing oxygen consumption profiles of FHF- and SHF-like CMs. **(D)**, Quantification of respiratory parameters in FHF- and SHF-like CMs. **(E)**, Immunocytochemical analyses for mitochondrial TOMM20 (red) in FHF-like CMs. Scale bar = 10 μm. **(F)**, Immunocytochemical analyses for mitochondrial TOMM20 (red) in SHF CMs. Scale bar = 5 μm. White and blue arrows denote punctate and filamentous mitochondria, respectively.

So far, our study identified an increase in sarcomeric organization and cellular stiffness, a decrease in migratory properties, and prolonged phase 2 plateau (APD_30_) of FHF compared to the SHF population. These results suggest that FHF population may represent a more mature state of CMs when compared to SHF cells. Another important parameter in CM maturation is the switch from glycolytic to fatty acid metabolism, with an increase in aerobic respiratory demand. Not surprisingly, our GO term analysis of top genes upregulated in FHF compared to SHF CMs showed an enrichment of GO:0006119: oxidative phosphorylation. Correspondingly, two independent gene expression analyses (scRNA and bulk RNA-seq) revealed that expression of key metabolic-related genes such as *CHCHD10, ATP5F1D, COX5A, and COX5B* were higher within the FHF compared to SHF population (Figures 4A,B).

To gain more insight into this process, we used respirometry to measure the levels of oxidative phosphorylation in the FHF and SHF populations. We performed a Seahorse Mitochondrial Stress Test using a Seahorse Extracellular Flux Analyzer to measure functional differences in respiration between FHF and SHF populations and found that the FHF- and SHF-like CMs show nearly identical respiratory profiles (Figure 4C). Among the respiratory parameters we measured with the mitochondrial stress test, we detected a significant difference only in oxygen consumption rate (OCR) contributed by ATP generation linked respiration for which FHF-like CMs showed greater OCR than SHF cells (Figure 4D). Additionally, we measured extracellular acidification rate (ECAR) from the same mitochondrial stress test as a proxy for glycolytic rate and found no significant differences between the FHF- and SHF-like CMs (Supplementary Figures S5A,B). Together, these data suggest that the FHF and SHF lineages share a common metabolic phenotype.

Finally, we visualized mitochondrial networks in FHF and SHF populations by confocal microscopy using antibodies against dsDNA and TOMM20. In both cell populations, we observed punctate mitochondrial networks (white arrows) with low nucleoid abundance. However, the FHF mitochondrial networks showed qualitatively higher numbers of filamentous mitochondrial (blue arrows) compared to the SHF networks (Figures 4E,F). In conclusion, our measurements suggest that the mitochondrial function and network morphology of FHF- and SHF-like CMs are highly similar despite the differences we observe in the transcription of several key metabolic genes.

### Pseudotime Analysis Shows Closer Developmental Progression of FHF Population to Human Fetal CMs

Our findings thus far suggest that expression of TBX5 in hESC-derived CMs may play a role in their maturation process, as we observed more organized sarcomeric structure and stiffness along with enhanced calcium ion signaling in FHF-like CMs, characteristics previously attributed to more mature CMs (Jiang et al., 2018; Guo and Pu, 2020; Karbassi et al., 2020). Despite recent efforts, *in vitro* differentiation strategies do not yield mature CMs that exhibit similar phenotypes to their adult endogenous counterparts. To understand where our hESC-generated CMs are positioned on the trajectory of cardiomyocyte development, we compared our CM populations with human CMs isolated from fetal hearts at three timepoints; 6, 10, and 17 weeks of gestation. We isolated single fetal cardiac cells (6 wks: *n* = 1,048, 10 wks: *n* = 3,779, and 17 wks: *n* = 2,948) for scRNA-seq using the 10X Genomics platform (Supplementary Figure S5A). We identified the CM cluster based on CM related genes such as *TNNT2* and *MYH7.* To avoid any contamination of other cells, we confirmed low or absent expression of fibroblast (*DDR2*, *COL1A1*) and endothelial markers (*CDH5*, *PECAM1*) within the isolated population (Supplementary Figure S5B). In addition, expression of key structural, calcium handling, and metabolic genes within these three fetal populations showed progressive increase with gestational age, confirming the influence of these pathways in the cardiac maturation process (Supplementary Figure S5C).

**FIGURE 5:**
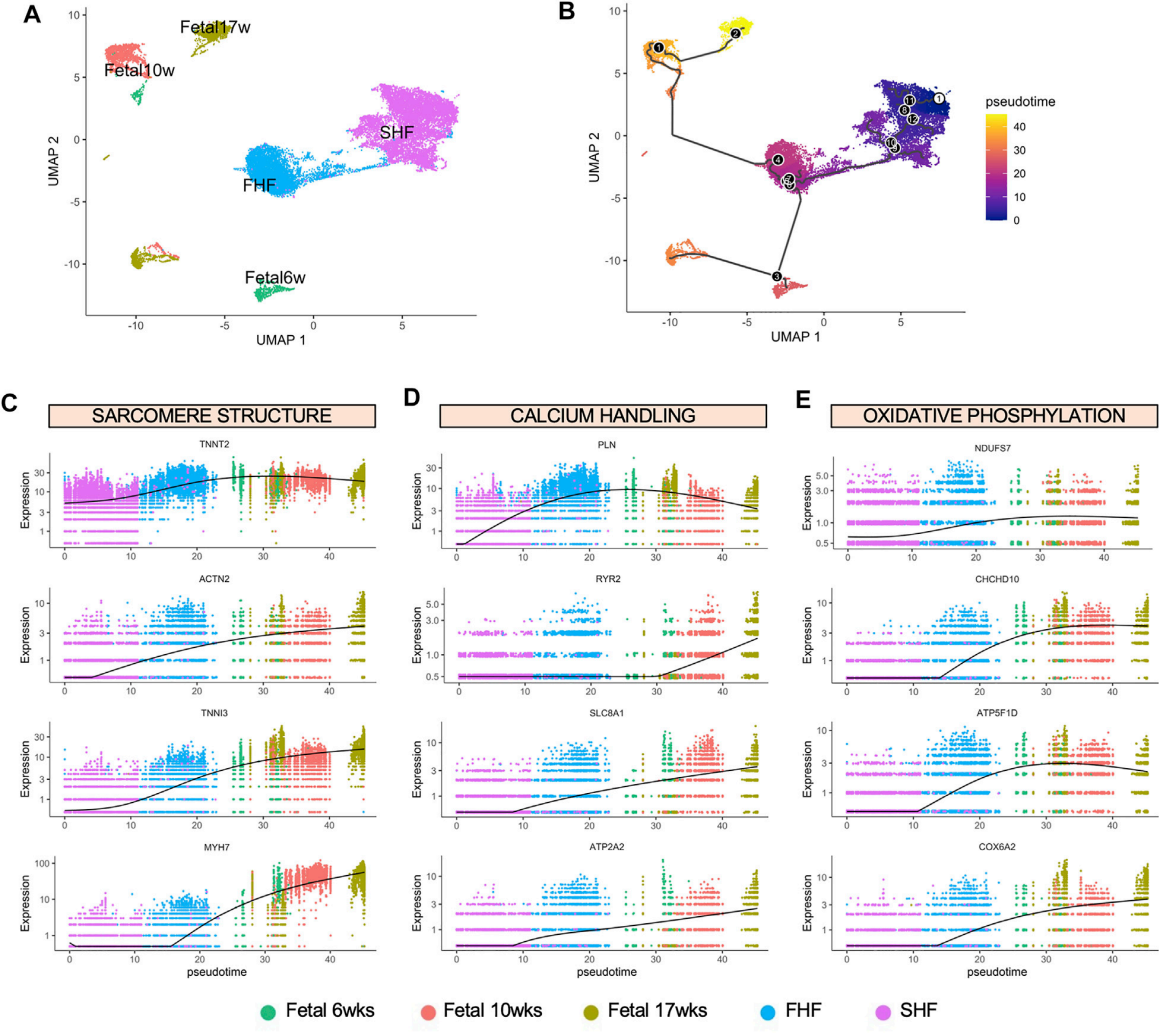
Monocle pseudotime analysis of FHF- and SHF-like CMs with human fetal CMs at different gestational age. **(A)**, UMAP showing clusters of hESC-derived CMs and human fetal CMs. **(B)**, Ordering of hESC-derived and human fetal CMs based on pseudotime, constructed by Monocle. Black circles indicate branch points. Dynamics of expression over pseudotime of key genes from GO Term analyses relating to **(C)**, sarcomere structure, **(D)**, calcium handling, and **(E)**, oxidative phosphorylation.

To determine the maturity level of FHF- and SHF-like CM populations in comparison to human fetal CMs, we integrated these populations together into one large dataset for Monocle trajectory analysis (Trapnell et al., 2014). Confirming our prior analysis using Seurat, FHF- and SHF-like CMs constitute two distinct clusters as shown by UMAP analysis (Figure 5A). We then aligned these five populations in pseudotime to determine the relative developmental trajectory of our hESC-derived CM populations (Figure 5B). Not surprisingly, we found that FHF-like CMs aligned in closer proximity to the fetal CM populations compared to SHF CMs. Pseudotime analyses of key genes from each pathway (structural: *TNNT2*, *ACTN2*, *TNNI3*, *MYH7*; calcium handing: *PLN*, *RYR2*, *SLC8A1*, *ATP2A2*; metabolism: *NDUFS7*, *CHCHD10*, *ATP5F1D*, *COX6A2*) confirmed not only the closer alignment of FHF-like CMs to the fetal CM populations, but also higher overall expression of these genes compared to SHF CMs (Figures 5C–E). These findings further emphasize the important role of TBX5 expression in regulating the *in vitro* maturation process, particularly in structural and calcium handling pathways.

## Discussion

The heart is the first functional solid organ during embryogenesis. In early embryonic development, mesodermal cells under the influence of secreted morphogens such as BMP4, Wnts and Activin A form the primitive streak (Galdos et al., 2017). Cardiac mesodermal cells migrate anterolaterally to form the cardiac crescent that gives rise to the primitive heart tube (Lescroart et al., 2014). These migratory cells include two heart-field specific progenitors known as FHF and SHF (Bruneau, 2013; Kelly et al., 2014). FHF progenitors mainly differentiate into CMs as they give rise to the left ventricle and part of atria (Bruneau et al., 1999). In contrast, SHF progenitors participate in elongation and looping of the heart tube, and because of their role, are highly proliferative and migratory. As these cells enter the heart tube to form the right ventricle, outflow tract, and atria, they differentiate into multiple cardiac cell types, such as CMs and smooth muscle cells (Kelly et al., 2014). Previous studies investigated the transcriptional profiles of these two populations and revealed that SHF progenitors are marked by the expression of *TBX1*, *FGF8*, *FGF10* and *SIX2* whereas FHF cells express *HCN4* and *TBX5* (Huynh et al., 2007; Watanabe et al., 2012; Später et al., 2013; Zhou et al., 2017).

Given the limited ability of adult CMs to proliferate in response to injury, much of effort has been focused on using human pluripotent stem cells to generate exogenous sources of CMs for cardiac cell-based regenerative therapy (Garbern and Lee, 2013). Our team (Pezhouman et al., 2021) and Zhang et al. (2019), for the first time, were able to isolate and characterize heart-field specific CMs from hESC and iPSCs, respectively. This is an important step in understanding early cardiac developmental processes as well as safer cell-based regenerative medicine, as prior transplantation studies used a heterogenous CM population which led to arrythmias (Liu et al., 2018; Romagnuolo et al., 2019). These prior studies focused on first isolating different subpopulations of CMs and nodal cells based on their expression of TBX5 or NKX2-5. This is followed by confirmation of their identify by profiling the expression of well-established transcriptional factors within each subpopulation. Although our previous study enabled us to separate FHF- from SHF-like CMs, using *TBX5* and *NKX2-5* expression, the role of TBX5 and its effects on underlying gene regulatory networks driving phenotypical and functional differences between these two populations remains unknown.

To uncover the underlying regulatory networks, we utilized techniques that enable rapid and unsupervised profiling of thousands of individual cells at the transcriptional, translational, and functional level, providing a more reliable representation of the differences between these two populations. First, we used scRNA-seq to obtain differentially expressed genes between FHF- and SHF-like CMs at the transcriptional level. Gene ontology analyses yielded four main biological processes including 1) muscle cell development (sarcomere structure), 2) oxidative phosphorylation (metabolism), 3) regulation of cardiac muscle contraction by calcium ion signaling (Ca^2+^ handling) and 4) response to wound healing (migration). We then used a variety of techniques such as Quantitative phase imaging, Seahorse assay, and Optical mapping to correlate gene expression differences to their structural and functional profiles.

Our structural analyses showed more mature sarcomeric organization in FHF- compared to SHF-like CMs, even in the presence of prolonged culture (i.e., up to 60 days), suggesting that intrinsic differences between these two populations may be the main drivers. Although the intrinsic differences can be attributed to many different processes, two categories that are known to regulate structural organization of CMs are genes that are directly involved in the formation of the sarcomeric unit (*TTN*, *NEBL*, *MYOM1*, and *ACTN2*) as well as genes involved in the regulation of the actin-myosin binding interactions (*MYL3*, *MYL4*, *MYH6*, and *MYH7*). The increased expression of these genes within FHF population can be associated with a higher degree of cellular stiffness we observed using atomic force microscopy. Prior studies have shown that cardiac muscle lacking nebulin (NEBL) resulted in variable lengths of thin filaments and lower isometric tension (Robinson and Winegrad, 1977; Robinson and Winegrad, 1979; Burgoyne et al., 2008) and that mutations in titin (TTN) can strongly affect cardiac muscle function due to changes in length-dependent activation of cross-bridges (Robinson and Winegrad, 1977; Robinson and Winegrad, 1979; Cazorla et al., 2001; Burgoyne et al., 2008; Granzier et al., 2009; Chung et al., 2013; Methawasin et al., 2014). Studies have also suggested that cardiac myofibrils are stiffer than skeletal myofibrils because Z-bands, titin filament networks, and other components of sarcomere structures within cardiac myofibrils are stronger than those of skeletal muscle (Akiyama et al., 2006). These data support our findings of increased expression and alignment of *ACTN2* and *MYOM1* in the FHF population, which forms the Z- and M-bands, respectively (Akiyama et al., 2006). Taken together, these data suggest that a downstream effect of TBX5 expression is the enhanced alignment and sarcomeric length regulation which leads to increased force generation, requisite for proper function of CMs from the left ventricle, primarily derived from the FHF population.

In addition to structural genes, our GO term analyses revealed differences in calcium signaling between the FHF and SHF populations. The action potential is central to CM function because it not only initiates but also regulates and coordinates excitation-contraction coupling. The morphology of APs reflects the net balance among ionic currents across the cell membrane. Among these ionic currents, calcium (Ca^2+^) is a critical regulator of CM function and predominantly contributes to phase 2 (plateau) of AP morphology**.** Regulation of calcium current within CMs are mediated by a multitude of voltage channels and regulatory proteins such as LTCC, RYR2, SERCA, PLN, and TRDN. In alignment with our findings of enhanced sarcomeric organization, we observed higher expression of these key calcium handling genes within the FHF compared to SHF population.

Given the key role of calcium in excitation-contraction coupling, it is not surprising that subtle changes in these components can have profound consequences on AP plateau (which can be measured by APD_30_) and contraction duration of CMs. To accurately quantify differences in AP plateau between our FHF and SHF population, we used a high-resolution imaging system (optical mapping) which allowed us to investigate the electrophysiological properties of thousands of cells within a large population at once. Our functional studies showed prolonged APD_30_ in FHF-like CMs consistent with our gene expression analyses. The increase in APD_30_ can be, in part, attributed to higher expression of PLN, which exhibits an inhibitory effect on SERCA, leading to decreased re-uptake of intracellular calcium and prolonging the duration of AP depolarization (Fearnley et al., 2011).

As CMs differentiate and mature in the developing embryo, there are dramatic changes in energy sources and metabolism. During early cardiac development, glycolysis is a major source of energy for CM migration, proliferation, maturation, and contraction. As these cells mature, there is a switch from glycolytic to oxidative metabolism to support the increase in metabolic needs of functional CMs (Gaspar et al., 2014). This switch enables CMs to metabolize different carbon sources such as fatty acids, ketone bodies, and branched-chain amino acids to maintain cardiac function despite changes in substrate availability (Lloyd et al., 2004; Kolwicz et al., 2013). While our GO term analyses revealed potential metabolic differences between FHF- and SHF-like CMs, assessment of multiple aspects of oxidative phosphorylation revealed nearly identical respiratory profiles, albeit an increase in ATP-linked OCR within FHF-like CMs. There are a couple potential reasons that can be attributed to these findings. First, despite observing differences in structural proteins and calcium handling within FHF compared to SHF CMs, it is very likely that these two populations, having been derived *in vitro*, have not surpassed a maturation state beyond the embryonic period when compared to their *in vivo* counterparts. For this reason, both populations are still relatively immature and predominantly rely on glycolytic pathways. Indeed, prior work have shown that in contrast to their fetal counterparts, hiPSC-CMs have deficient fatty acid oxidation (FAO) despite expression of appropriate genes (Hui Zhang et al., 2020). Second, *in vitro* cardiac differentiation relies on specific media that is lipid-poor and glucose-rich (RPMI-B27) which has been shown to suppress FAO and may prevent the metabolic switch from glycolysis to oxidative phosphorylation (Saggerson, 2008; Lian et al., 2012; van Weeghel et al., 2018). Our study did not identify a role of TBX5 or its downstream mediators in coordinating the metabolic switch of hESC-derived CMs, although further studies are needed.

Despite uncovering differences between FHF and SHF populations, our study is limited by the use of *in vitro* hESC-derived CMs. Countless efforts to promote the maturation states of hESC-derived CMs have been unsuccessful thus far. Studies have shown that these cells are more similar to human embryonic CMs rather than their adult counterparts (Snir et al., 2003; Lieu et al., 2009). As our study yielded differences in sarcomeric structure and calcium handling suggestive of increased maturation in FHF compared to SHF CMs, we turned to human fetal heart samples and Monocle analyses to determine the proximity of FHF- and SHF-like CMs to endogenous fetal CMs along on the normal human cardiac development trajectory. Not surprisingly, trajectory analyses showed that SHF CMs were aligned earliest in pseudotime and that increased expression of sarcomeric proteins and calcium handling within FHF-like CMs resulted in a shift of this population farther along the development trajectory. As expected, these two populations lagged behind the human fetal heart samples on the trajectory inference, which were generally aligned accordingly to developmental age.

The role of TBX5 expression in cardiac development and heart field specification has been studied in detail. Here, we report isolation of FHF- and SHF-like CMs by differentiating our double reporter hESC line to uncover pivotal roles of TBX5 and its downstream effects on cardiac maturation. These effects include increased expression of key sarcomeric structure genes as well as calcium handling machinery. While metabolic differences may be attributable to *TBX5* expression, our study did not show respiratory distinctions between these two populations. These findings pave the way for further investigations into the modulation of *TBX5* expression as a potential method to regulate *in vitro* cardiac maturation which can serve as platforms for deeper understanding of the cardiac maturation process as well as development of more effective cardiac regenerative therapies.

## Data Availability

The datasets presented in this study can be found in online repositories. The names of the repository/repositories and accession number(s) can be found in the article/Supplementary Material.

## Supplementary Material

Supplementary Figure S1 Differentiation and isolation of FHF- and SHF-like CMs for scRNA-seq analysis. **(A)**, Schematic of the insertion of TdTomato and eGFP into the *TBX5* (Exon 9) and *NKX2-5* (Exon1) loci in previously generated hESC *TBX5-TdTomato* ^*+/W*^/*NKX2-5* ^eGFP/W^ double reporter line. **(B)**, Schematic of monolayer cardiac differentiation protocol. **(C)**, Fluorescence microscopy of FHF (TdT^+^/GFP^+^) and SHF (TdT^-^/GFP^+^) optimized cardiac differentiations, scale bar = 100 μm. **(D)**, Representative flow cytometric analysis at Day 20 of cardiac differentiation showing the expression of TBX5 and NKX2-5. **(E)**, Top genes associated with principal components 1 and 2. **(F)**, DimPlot of FHF and SHF populations based on principal components 1 and 2. Hypergeometric tests show significant **(G)**, *TBX5* and **(H)**, *NKX2*-5 occupancy at promoters of murine FHF and SHF gene orthologs when compared to 10 random sets of 150 genes that do not include FHF nor SHF genes (y-axis shows -log_10_(corrected p-value)). bioChIP-seq peaks from murine ventricles (E12.5) were used to define regions of *TBX5* or *NKX2*-5 occupancy in the mm9 genome (Akerberg et al., 2019). Red dashed line crosses the y-axis at −log_10_(0.05) as a reference point.

Supplementary Figure S2 Differences in sarcomeric structure and cell migration between FHF- and SHF-like CMs. **(A)**, Boxplots of cardiac sarcomeric structural genes extracted from GO pathway “GO:0030049: muscle filament sliding” with higher average expression in SHF compared to FHF-like CMs. **(B)**, Heatmap of genes from panel A using bulk RNA sequencing data of hESCs and FHF- and SHF-like CMs at Day 20. **(C)**, Boxplots of migratory related-genes extracted from GO pathway “GO:0009611: response to wounding” with higher average expression in SHF compared to FHF-like CMs. **(B)**, Heatmap of genes from panel C using bulk RNA sequencing data of hESCs and FHF- and SHF-like CMs at Day 20. **(E)**, Quantitative phase imaging combined with fluorescent microscopy to identify and trace FHF-like (TdT^+^/GFP^+^) and SHF-like (TdT^-^/GFP^+^) CMs. Scale bar = 20 μm.

Supplementary Figure S3 Transcriptional and functional analyses of calcium handling machinery in FHF- and SHF-like CMs. **(A)**, Boxplots of calcium handling-related genes extracted from GO term “GO:0010882: regulation of cardiac muscle contraction by calcium ion signaling” with higher average expression in SHF compared to FHF-like CMs. **(B)**, Heatmap of genes from panel A using bulk RNA sequencing data of hESCs and FHF- and SHF-like CMs at Day 20. **(C)**, APD_30_ (left) and APD80 (right) histograms of APs recorded from FHF-like CMs (*n* = 7,776 pixels). **(D)**, APD_30_ (left) and APD80 (right) histograms of APs recorded from SHF CMs (*n* = 8,464 pixels).

Supplementary Figure S4 Extracellular acidification rate analyses in FHF- and SHF-like CMs. **(A)**, Seahorse assay measuring extracellular acidification rate (ECAR) profiles of FHF- and SHF-like CMs. **(B)**, Quantification of ECAR parameters between FHF-like and SHF-like CMs.

Supplementary Figure S5 Single cell RNA sequencing analysis of human fetal CMs. **(A)**, UMAP showing clusters of 6, 10, and 17 weeks human fetal CMs. **(B)**, UMAP showing gene expression quantification for the cardiac genes (*TNNT2, MYH7)*, fibroblast genes (*DDR2*, *COL1A1*), and endothelial genes (*CDH5, PECAM1*). **(C)**, Violin plots of expression of key genes from GO Term analyses relating to sarcomere structure, calcium handling, and oxidative phosphorylation.
