# Supplementary figures and images for: Transcriptional, Electrophysiological, and Metabolic Characterizations of hESC-Derived First and Second Heart Fields Demonstrate a Potential Role of TBX5 in Cardiomyocyte Maturation
Source: Front Cell Dev Biol. 2021 Dec 17;9:787684. doi: 10.3389/fcell.2021.787684 (PMC8722677; doi:10.3389/fcell.2021.787684)

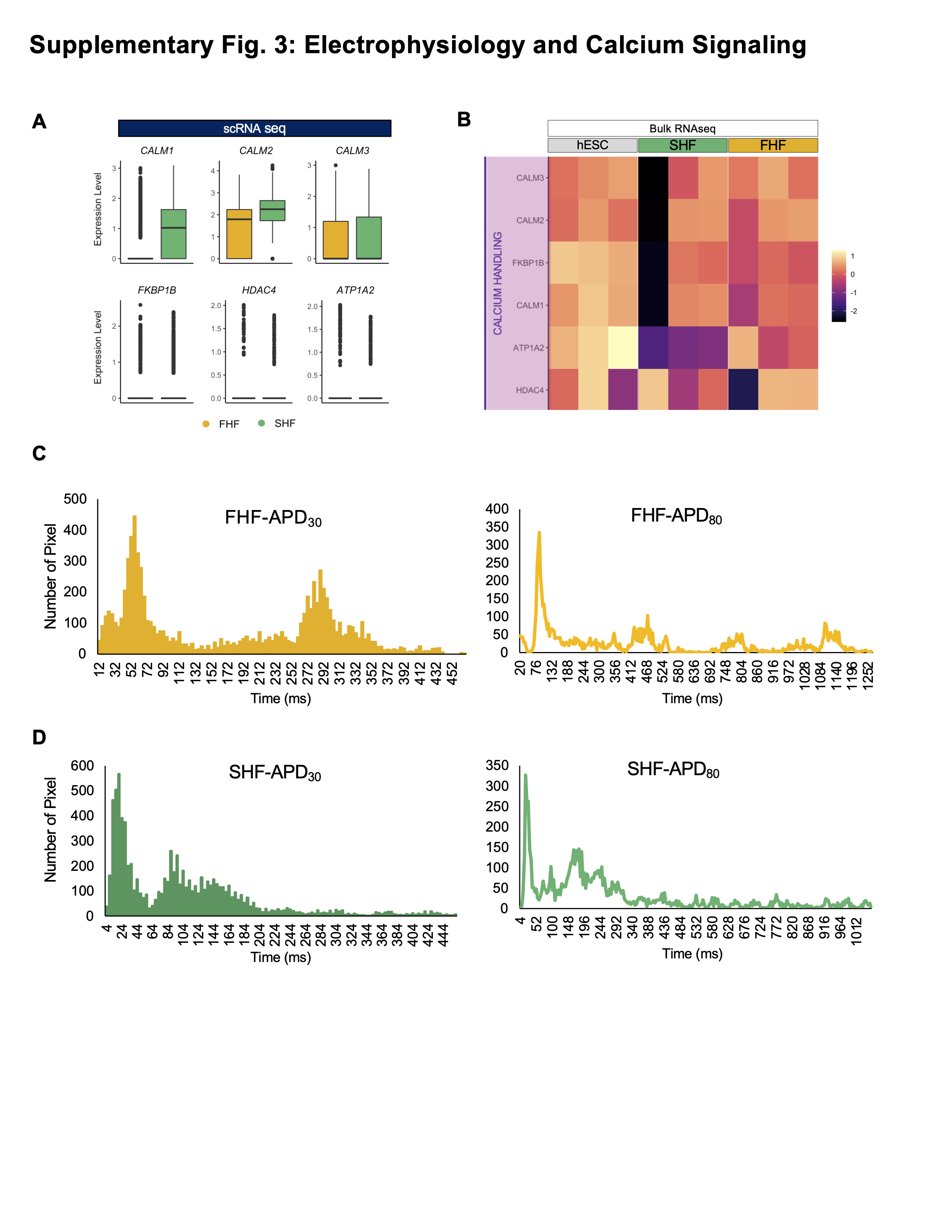

Supplement: Supplementary file 1 [file Image3.tiff]

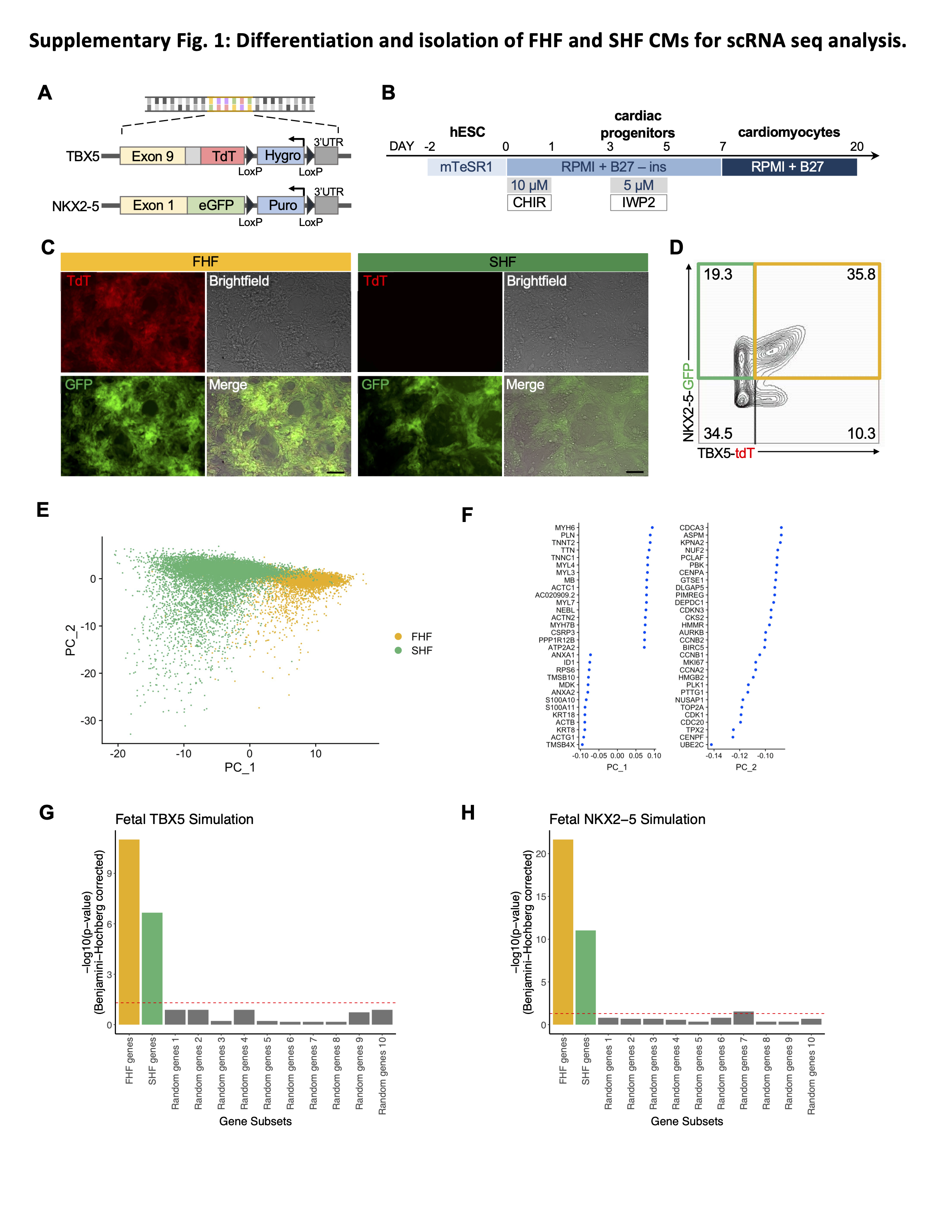

Supplement: Supplementary file 2 [file Image1.tiff]

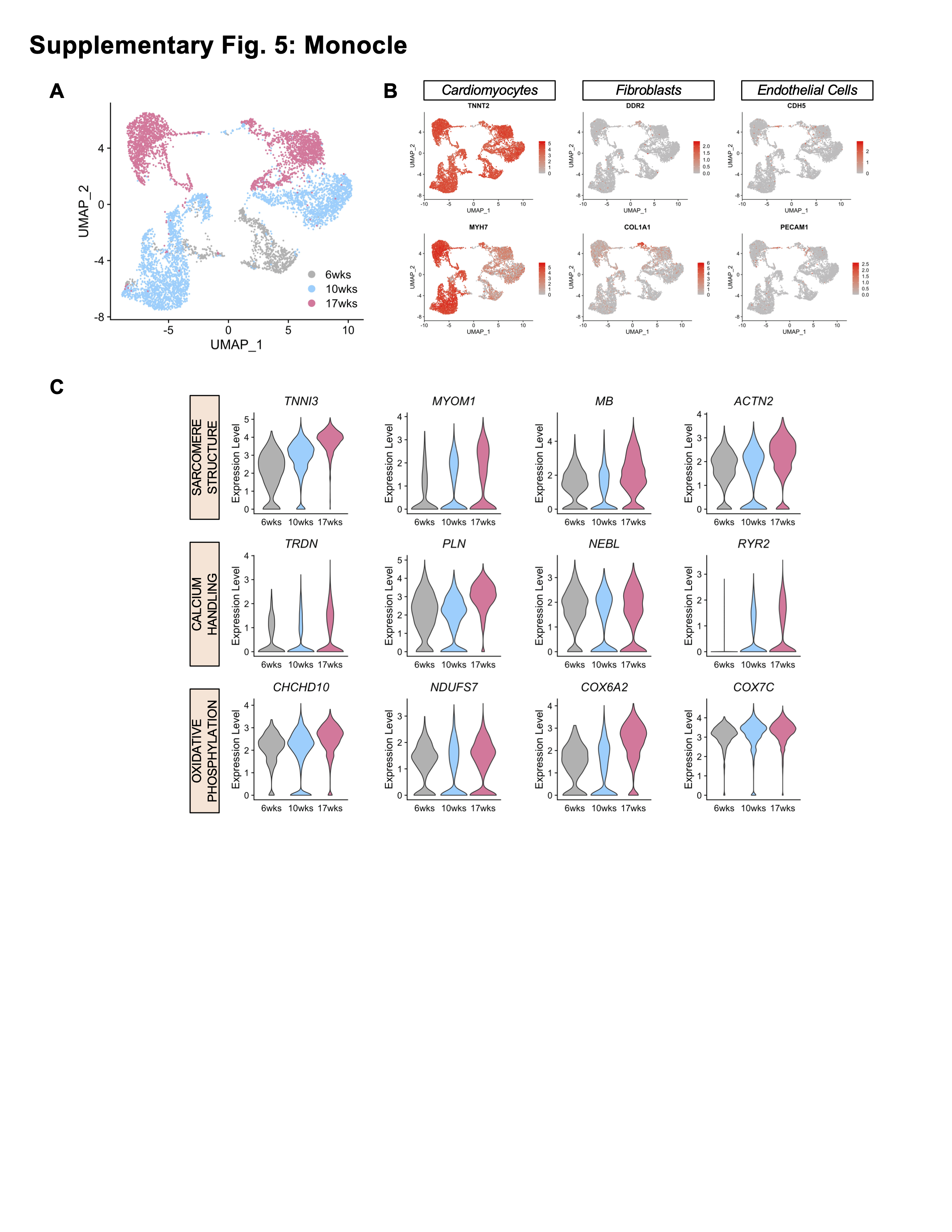

Supplement: Supplementary file 3 [file Image5.tiff]

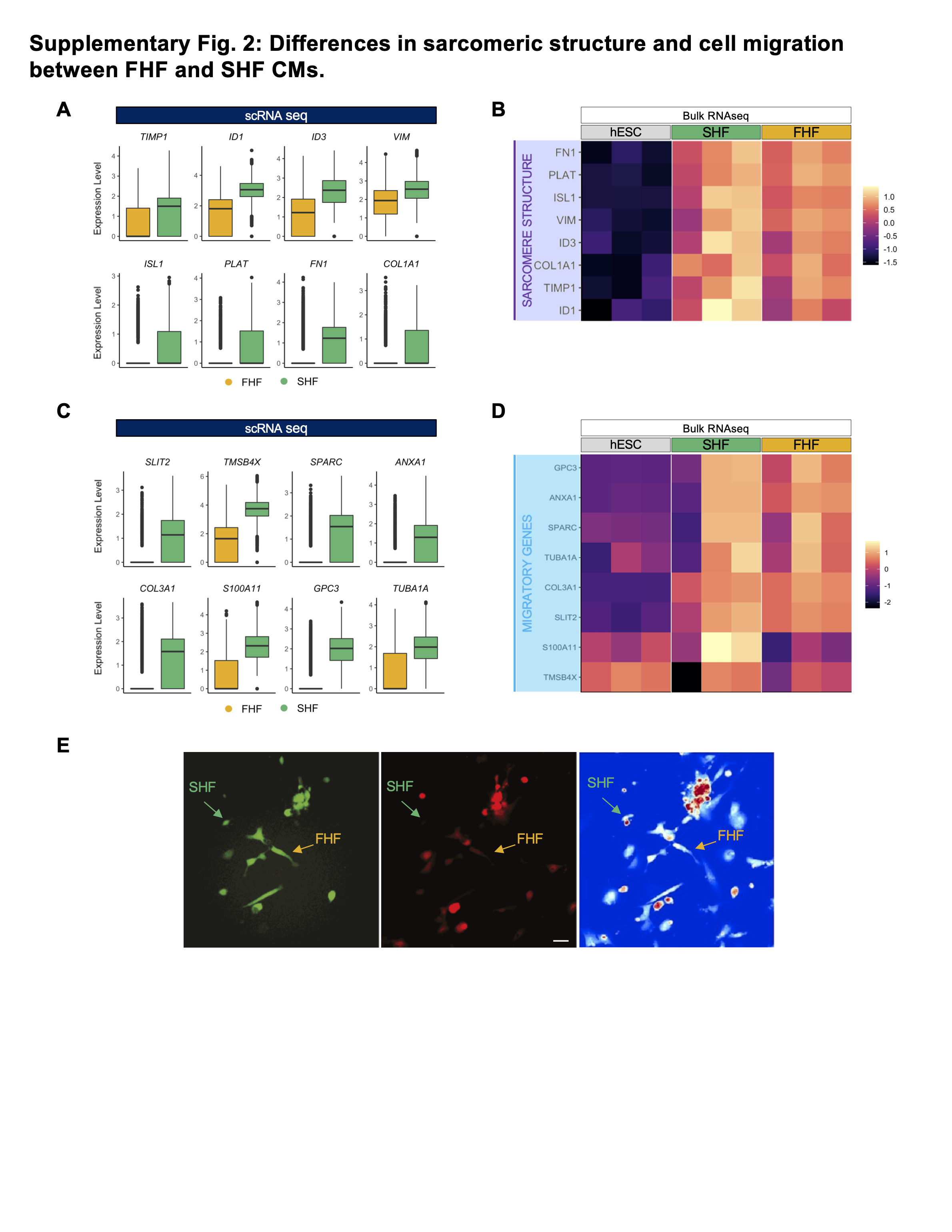

Supplement: Supplementary file 4 [file Image2.tiff]

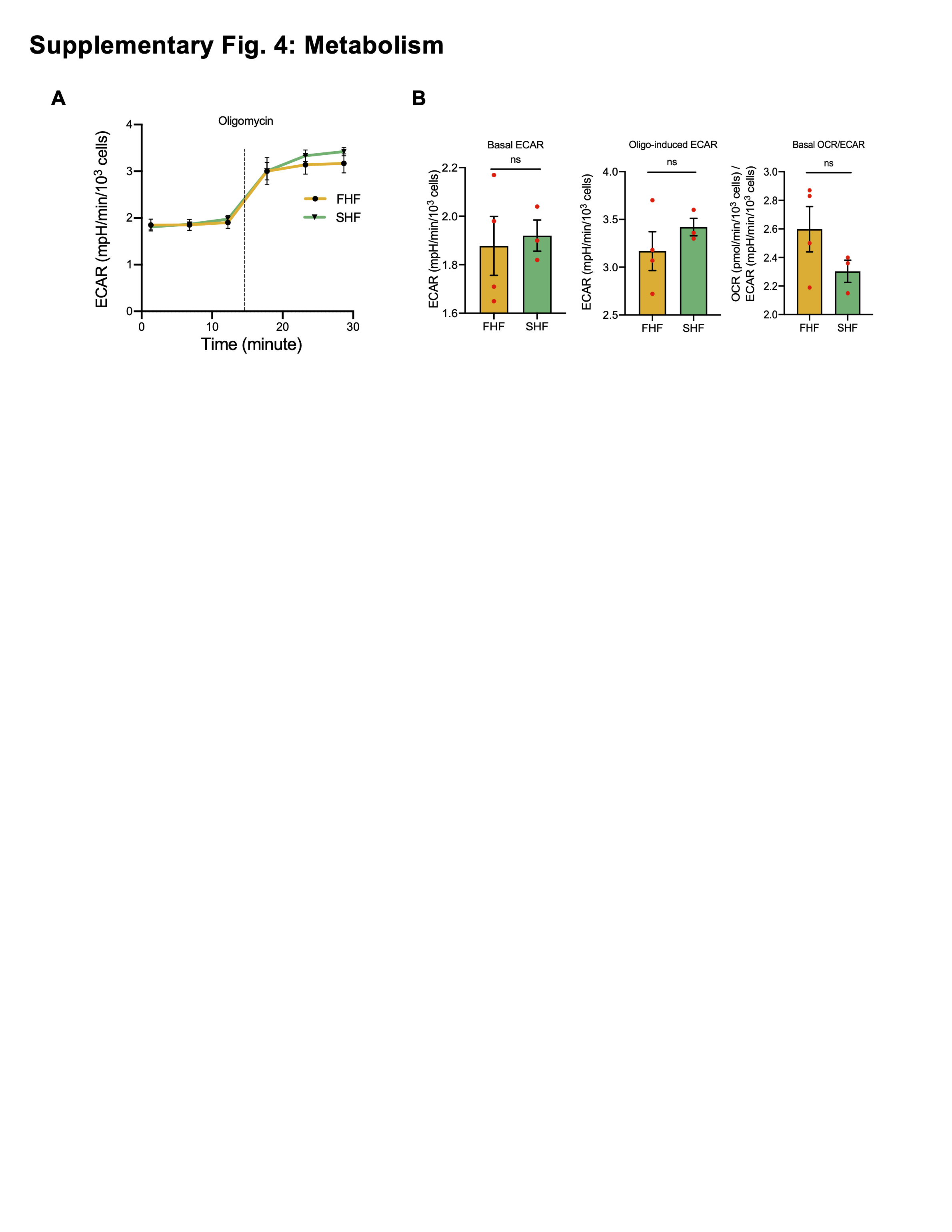

Supplement: Supplementary file 5 [file Image4.tiff]
